# Association between single nucleotide polymorphism rs9534275 and the risk of coronary artery disease and ischemic stroke

**DOI:** 10.1186/s12944-017-0584-5

**Published:** 2017-10-05

**Authors:** Liu Miao, Rui-Xing Yin, Shuo Yang, Feng Huang, Wu-Xian Chen, Xiao-Li Cao

**Affiliations:** 1 Department of Cardiology, Institute of Cardiovascular Diseases, The First Affiliated Hospital, Guangxi Medical University, 22 Shuangyong Road, Nanning, 530021 Guangxi People’s Republic of China; 2 Department of Neurology, The First Affiliated Hospital, Guangxi Medical University, 22 Shuangyong Road, Nanning, 530021 Guangxi People’s Republic of China

**Keywords:** Breast susceptibility gene 2, Single nucleotide polymorphism, Lipids, Coronary artery disease, Ischemic stroke

## Abstract

**Background:**

The present study was to detect the association of single nucleotide polymorphism (SNP) in the breast susceptibility gene 2 (*BRCA2*) and the risk of coronary artery disease (CAD) and ischemic stroke (IS).

**Methods:**

Genotypes of the *BRCA2* rs9534275 in 1822 unrelated subjects (CAD, 606; IS, 569; and healthy controls, 647) were determined by the polymerase chain reaction and restriction fragment length polymorphism and then confirmed by direct sequencing.

**Results:**

The genotypic and allelic frequencies of rs9534275 were significantly different between the CAD, IS patients and controls (*P* = 0.033 and *P =* 0.027; respectively). The GG, GT/GG genotypes and G allele were associated with an increased risk of CAD and IS (CAD: *P* = 0.005 for GG *vs.* TT, *P =* 0.004 for GT/GG *vs.* TT, *P* = 0.005 for G *vs.* T; IS: *P* = 0.003 for GG *vs.* TT, *P* = 0.005 for GT/GG *vs.* TT; *P* = 0.002 for G *vs.* T). The GG, GT and GT/GG genotypes in the CAD, but not in healthy controls and IS patients, were associated with an increased serum total cholesterol (TC) and apolipoprotein B (ApoB) concentration.

**Conclusions:**

The present study shows that the G allele carriers of *BRCA2* rs9534275 were associated with increased serum TC and ApoB levels in the CAD patients and increased risk of CAD and IS.

**Trial registration:**

Retrospectively registered.

## Background

Both coronary artery disease (CAD) and ischemic stroke (IS) are the major causes of morbidity and death in the developed countries, and are also the leading cause of long-term disability in survivors [1, 2]. Atherogenic dyslipidemia characterized by low levels of high-density lipoprotein cholesterol (HDL-C) and apolipoprotein (Apo) A1, high levels of total cholesterol (TC), triglyceride (TG) and low-density lipoprotein (LDL) particle number is highly associated with increased incidence of the cardiovascular disease [3] and IS [4, 5]. In addition, genetic factors are estimated to account for about 50–80% of the variation in serum lipid levels [6], and 30–60% of the incidence of CAD and IS [7]. Therefore, single nucleotide polymorphisms (SNPs) in the lipid-related genes may have some associations with serum lipid levels, and the risk of CAD and IS [8].

A few previous GWASes have proved that the breast cancer susceptibility gene 2 (*BRCA2*; Also knows as: *FAD; FACD; FAD1; GLM3; BRCC2; FANCD; PNCA2; FANCD1; XRCC11; BROVCA2*, Gene ID: 675, HGNC ID: 1101, synonyms: “*BRCA1/BRCA2*-containing complex, subunit 2”, *BRCC2, FAD, FAD1, XRCC11*, locus type: gene with protein product, chromosomal location: 13q13.1) mutation can cause an increased risk for breast cancer [9]. Women carrying *BRCA* mutations have metabolic deregulations in their breast tissue that may be precursors to malignant transformation, and also lead to exhibit a reduction of 79% in metabolite level, while both lipid unsaturation and TG levels increased by 19%. Besides these, women carrying *BRCA2* mutations showed an increased lipid unsaturation of 21% and the metabolic changes in women carrying *BRCA1* mutations were different from those in women carrying *BRCA2* mutations, with a 47% increase in cholesterol level recorded in those with *BRCA2* mutations [10]. The mechanism was supposed to have a connection with lipid metabolism [11]. A previous GWAS on plasma lipid levels has identified the rs9534275 SNP near the *BRCA2* as hyperlipidemic locus in European. And, several previous studies have shown that the *BRCA2* rs9534275 may have an effect on TC, low-density lipoprotein cholesterol (LDL-C), and serum lipid levels might have ethnic- and/or sex-specificity [12, 13].

To our knowledge, the genetic evidence on the association between *BRCA2* variants and atherosclerosis in humans is poor. In a previous study, we have found that the *BRCA2* rs9534275 SNP modulated serum TC, LDL-C, ApoB concentrations, and the ApoA1/ApoB ratio in the hypercholesterolemic subjects [14], suggesting that the rs9534275 SNP plays an important role in the formation of atherosclerosis. Therefore, the present study aimed to determine whether the *BRCA2* rs9534275 SNP is associated with the risk of CAD and IS in the Guangxi Han population.

## Methods

### Subjects

A total of 606 patients with CAD and 569 patients with IS were recruited from hospitalized patients in the First Affiliated Hospital, Guangxi Medical University. All of the enrolled CAD patients were evaluated by coronary angiography due to suspected CAD or unrelated conditions requiring angiographic evaluation; the coronary angiograms were analyzed by two experienced interventional cardiologists. CAD was defined as significant coronary stenosis (≥ 50%) in at least one of the three main coronary arteries or their major branches (branch diameter ≥ 2 mm). Subjects with congenital heart disease and type I diabetes mellitus were excluded [15]. All of the enrolled IS patients received a strict neurological examination and brain magnetic resonance imaging. The diagnosis of IS was according to the International Classification of Diseases (9th Revision). Patients with a transient ischemic attack, embolic brain infarction, stroke caused by inflammatory disease, cardio embolic stroke, autoimmune disease, or serious chronic diseases were excluded from this study. Subjects with a past history of CAD were also excluded from the study [16]. A total of 647 healthy controls matched by age, gender, and geographical area were included. The controls were judged to be free of CAD and IS by questionnaires, medical history, and clinical examination. All individuals enrolled were from the Han population in Guangxi, China. A standard questionnaire was used to ascertain general information and medical history from all participants. The study protocol was approved by the Ethics Committee of the First Affiliated Hospital, Guangxi Medical University (No. Lunshen 2009-Guike-018; Jan. 7, 2009). Informed consent was obtained from all subjects after receiving a full explanation of the study [17].

### Genotyping and biochemical analysis

**Fig. 1:**
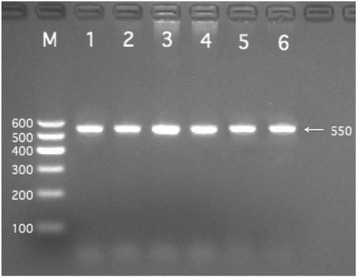
Electrophoresis of polymerase chain reaction products of the samples. Lane M is the 100 bp marker ladder; Lanes 1–6 are samples, the 550 bp bands are the target genes

**Fig. 2:**
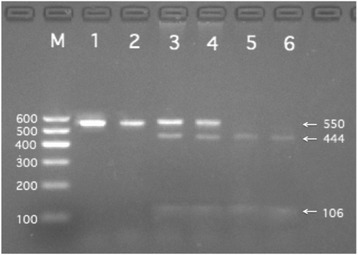
Genotyping of the *BRCA2* rs9534275 SNP. Lane M, 100 bp marker ladder; lanes 1 and 2, TT genotype (550 bp); lanes 3 and 4, GT genotype (444- and 106-bp); lanes 5 and 6, GG genotype (444- and 106-bp)

**Fig. 3:**
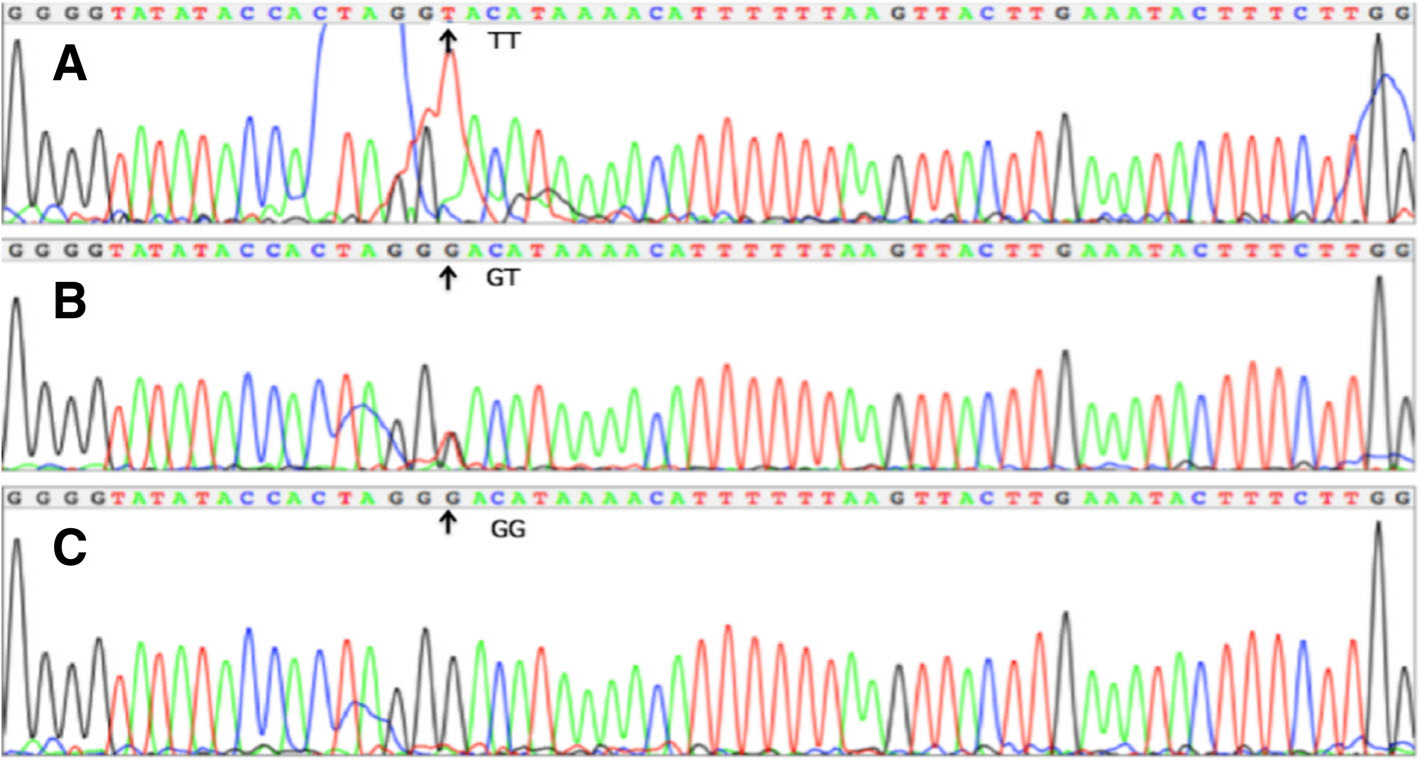
A part of the nucleotide sequence of the *BRCA2* rs9534275 SNP. **a**: TT genotype; **b**: GT genotype; and **c**: GG genotype

All of the biochemical assays and genotyping in CAD and IS patients were performed after hospitalization, and all of the venous blood samples were obtained from the patients and controls after at least 12 h of fasting. Genomic DNA was isolated from peripheral blood leukocytes using the phenol-chloroform method. Genotyping of the *BRCA2* rs9534275 was performed by polymerase chain reaction and restriction fragment length polymorphism (PCR-RFLP). PCR amplification was performed using 5′-TCTTGGCCCAGATGCTTACT-3′ as the forward and 5′-TACCAACACTACCACCAGCA-3′ as reversed primer pair (Sangon, Shanghai, People’s Republic of China), respectively. Each 25 μL PCR reaction mixture consisted of 2.0 μL genomic DNA, 1.0 μL each primer (10 μmol/L), 12.5 μL of 2 × *Taq* PCR Master mix (constituent: 0.1 U *Taq* polymerase/μL, 500 μM dNTP each and PCR buffer.), and 8.5 μL of ddH_2_O (DNase/RNase-free). PCR was performed with an initialization step of 95 °C for 5 min, followed by 30 s denaturing at 95 °C, 30 s of annealing at 59 °C and 35 s of elongation at 72 °C for 33 cycles. The amplification was completed by a final extension at 72 °C for 7 min. Following electrophoresis on a 2.0% agarose gel with 0.5 μg/mL ethidium bromide, the amplification products were visualized under ultraviolet light. Subsequently, each restriction enzyme reaction was performed with 5.0 μL amplified DNA, 8.8 μL nuclease-free water, 1.0 μL of 10 × buffer solution and 0.2 μL *Rsa*I restriction enzyme in a total volume of 15 μL digested at 37 °C overnight. After restriction enzyme digestion of the amplified DNA, genotypes were identified by electrophoresis on 2% ethidium-bromide stained agarose gels and visualized with UV illumination. An experienced reader blinded to the epidemiological and serum lipid results scored the genotypes. Six samples (TT, GT and GG genotypes in two (Figs. 1, 2 and 3; respectively) detected by the PCR-RFLP were also confirmed by direct sequencing with an ABI Prism 3100 (Applied Biosystems) in Shanghai Sangon Biological Engineering Technology & Services Co., Ltd., People’s Republic of China [18]. The levels of serum TC, TG, HDL-C, and LDL-C in the samples were determined by enzymatic methods with commercially available kits. Serum ApoA1 and ApoB levels were detected by an immunoturbidimetric immunoassay using a commercial kit [19, 20].

### Diagnostic criteria

The normal values of serum TC, TG, HDL-C, LDL-C, ApoA1, ApoB levels and the ApoA1/ApoB ratio in our Clinical Science Experiment Center were 3.10–5.17, 0.56–1.70, 0.91–1.81, 2.70–3.20 mmol/L, 1.00–1.78, 0.63–1.14 g/L, and 1.00–2.50; respectively. The individuals with TC > 5.17 mmol/L and/or TG > 1.70 mmol/L were defined as hyperlipidemic. Hypertension was diagnosed according to the criteria from the 1999 World Health Organization-International Society of Hypertension Guidelines for the management of hypertension [21]. The diagnostic criteria of overweight and obesity were according to the Cooperative Meta-analysis Group of China Obesity Task Force. Normal weight, overweight and obesity were defined as a BMI < 24, 24–28 and >28 kg/m^2^, respectively [22]. Dyslipidemia was defined according to World Health Organization criteria: TG ≥ 1.7 mmol/L and HDL-C < 0.9 mmol/L for men or <1.0 mmol/L for women. Diabetes was defined as a fasting plasma glucose ≥7.0 mmol/L or 2 h postprandial plasma glucose ≥11.1 mmol/L or as having been previously diagnosed with diabetes and receiving therapy [23].

### Statistical analyses

All statistical analyses were performed using the statistical software package SPSS 21.0 (SPSS Inc. Chicago, IL, USA). A standard goodness-of-fit test was used to test the Hardy-Weinberg equilibrium. A chi-square analysis was used to evaluate the difference in genotype distribution and sex ratio between the groups. The general characteristics between the cases and controls were tested using Student’s unpaired *t*-test. The association between genotypes and serum lipid parameters was tested by analysis of covariance (ANCOVA). Sex, age, body mass index (BMI), blood pressure, alcohol consumption, and cigarette smoking were adjusted for the statistical analysis. Odds ratio (OR) and 95% confidence interval (CI) were calculated using unconditional logistic regression. A two-tailed *P* value less than 0.05 was considered to be statistically significant.

## Results

### General characteristics and serum lipid levels

The baseline characteristics of the patients with CAD or IS and the controls are shown in Table 1. The mean age, male to female ratio, average BMI, serum TG, ApoA1, ApoB levels and the ApoA1/ApoB ratio and the percentages of subjects who smoked cigarettes were similar between the controls and CAD patients or between the controls and IS patients. The average height, weight, systolic blood pressure, pulse pressure, glucose, serum TC and LDL-C levels were significantly higher and serum HDL-C and the percentages of subjects who consumed alcohol were significantly lower in the CAD and IS patients than in the controls. The average diastolic blood pressure was lower in CAD patients, but higher in IS patients as compared with controls.

**Table 1:** Comparison of demographic, lifestyle characteristics and serum lipid levels of the participants

Characteristic	Control (*n* = 647)	Case		*P*_*vs.controls*_	
		CAD (*n* = 606)	IS (*n* = 569)	*CAD*	*IS*
Male/female	475/172	447/159	410/159	0.898	0.606
Age (years)	61.71 ± 9.60	62.21 ± 10.54	62.85 ± 12.33	0.596	0.451
Height (cm)	155.09 ± 7.82	164.10 ± 6.91	163.73 ± 7.27	0.026	0.042
Weight (kg)	54.55 ± 9.00	64.62 ± 10.63	63.08 ± 11.06	0.000	0.000
BMI (kg/m^2^)	22.66 ± 3.19	23.93 ± 3.22	23.47 ± 3.49	0.640	0.531
SBP (mmHg)	127.53 ± 18.71	132.89 ± 23.37	147.24 ± 22.30	0.000	0.000
DBP (mmHg)	80.29 ± 11.30	79.19 ± 13.24	83.62 ± 12.81	0.023	0.002
PP (mmHg)	47.23 ± 13.69	53.69 ± 17.18	63.63 ± 18.08	0.000	0.000
Smoking, n (%)	257(39.7)	272(44.9)	247(43.4)	0.067	0.200
Alcohol, n (%)	279(43.1)	155(25.6)	168(29.5)	0.000	0.000
Glu (mmol/L)	6.18 ± 1.76	6.38 ± 1.41	6.41 ± 1.52	0.004	0.000
TC (mmol/L)	4.50 ± 0.57	4.55 ± 1.20	4.53 ± 1.15	0.000	0.000
TG (mmol/L)	1.42 ± 1.74	1.67 ± 1.09	1.71 ± 1.53	0.747	0.277
HDL-C (mmol/L)	1.90 ± 0.48	1.15 ± 0.33	1.23 ± 0.40	0.000	0.000
LDL-C (mmol/L)	2.75 ± 0.72	2.96 ± 0.96	2.93 ± 0.84	0.000	0.000
Apo A1, g/L	1.41 ± 0.27	1.04 ± 0.53	1.02 ± 0.22	0.229	0.069
ApoB, g/L	0.91 ± 0.20	1.10 ± 4.56	0.99 ± 0.24	0.101	0.203
ApoA1/ApoB	1.64 ± 0.51	1.21 ± 0.50	1.09 ± 0.37	0.113	0.216

*SBP*, Systolic blood pressure; *DBP*, Diastolic blood pressure; *PP*, Pulse pressure; *Glu*, Glucose; *HDL-C*, high-density lipoprotein cholesterol; *LDL-C*, low-density lipoprotein cholesterol; *Apo*, Apolipoprotein. *TC*, Total cholesterol; *TG*, Triglyceride; The value of triglyceride was presented as median (interquartile range), the difference between the control and CAD/IS groups was determined by the Wilcoxon-Mann-Whitney test

### Genotypic and allelic frequencies

The frequency of the T and G alleles was 55.3% and 44.7% in the controls, 50.8% and 49.2% in the CAD patients (*P* = 0.025), and 50.2% and 49.8% in the IS patients (*P* = 0.012), respectively (Table 2). The frequency of the TT, GT and GG genotypes was 31.4%, 47.9% and 20.7% in the controls, 24.8%, 52.1% and 23.1% in the CAD patients (*P* = 0.033), and 24.6%, 51.1% and 24.3% in the IS patients (*P* = 0.027), respectively. The genotypic and allelic frequencies were concordant with those predicted by the Hardy-Weinberg proportions in both experimental groups (*P* = 0.330 for CAD and *P* = 0.620 for IS) and controls (*P* = 0.349).

**Table 2:** Genotype and allele frequencies of the *BRCA2* rs9534275 SNP in cases and controls

Genotype or allele	Control [*n* (%)]	CAD [*n* (%)]	IS [*n* (%)]	CAD		IS	
				OR (95% CI)	*P*	OR (95% CI)	*P*
TT	203 (31.4)	150 (24.8)	140 (24.6)	1		1	
GT	310(47.9)	316 (52.1)	291 (51.1)	1.58 (1.19–2.08)	0.365	1.52 (1.15–2.01)	0.214
GG	134 (20.7)	140 (23.1)	138 (24.3)	1.78 (0.82–1.91)	0.005	1.85 (1.32–2.59)	0.003
*X* ^*2*^		6.812	7.257				
*P*		0.033	0.027				
HWE (*P*)	0.349	0.33	0.62				
TT	203 (31.4)	150 (24.8)	140 (24.6)	1		1	
GT/GG	444 (68.6)	456 (75.2)	429 (75.4)	1.63 (1.25–2.12)	0.004	1.61 (1.24–2.10)	0.005
*X* ^*2*^		6.783	6.854				
*P*		0.01	0.009				
T	716 (55.3)	616 (50.8)	571 (50.2)	1		1	
G	578 (44.7)	596 (49.2)	567 (49.8)	1.35(1.14–1.60)	0.005	1.37 (1.16–1.62)	0.002
*X* ^*2*^		5.106	6.462				
*P*		0.025	0.012				

Adjusted for sex, age, smoking, drinking, BMI, diabetes, hypertension, hyperlipidemia. *CAD*, coronary artery disease; *IS*, ischemic stroke

### *BRCA2* rs9534275 SNP and the risk of CAD and IS

The G allele was associated with an increased risk of CAD (adjusted OR = 1.35, 95% CI = 1.14–1.60) and IS (adjusted OR = 1.37, 95% CI = 1.16–1.62; Table 2). The GG and GT/GG genotypes were also associated with an increased risk of CAD (adjusted OR = 1.78, 95% CI = 0.82–1.91 for GG vs. TT and adjusted OR = 1.63, 95% CI = 1.25–2.12 for GT/GG vs. TT) and IS (adjusted OR = 1.85, 95% CI = 1.32–2.59 for GG vs. TT and adjusted OR = 1.61, 95% CI = 1.24–2.10 for GT/GG vs. TT). Stratified analysis showed an increased risk of CAD in subjects with a GT/GG genotype, mainly in those who were part of one of the following groups: high BMI (adjusted OR = 1.47, 95% CI = 0.98–2.20), smokers (adjusted OR = 1.48, 95% CI = 1.03–2.14). There was an increased risk of IS in subjects with a GT/GG genotype, mainly in those who belonged to one of the following groups: high BMI (adjusted OR = 1.49, 95% CI = 0.97–2.23), smokers (adjusted OR = 1.90, 95% CI = 1.29–2.80) (Table 3). No significant interaction was detected between the genotypes and these factors.

**Table 3:** The risk of rs9534275 for CAD and IS according to body mass index (BMI), gender, smoking and drinking

Factors	Genetype	CAD			IS		
		OR (95% CI)	*P*	*P*_*interaction*_	OR (95% CI)	*P*	*P*_*interaction*_
BMI							
< 24 Kg/m^2^	TT GT/GG	1		0.480	1		0.560
		1.34(0.97–1.87)	0.078		1.36(0.97–1.87)	0.061	
≥ 24 Kg/m^2^	TT	1			1		
	GT/GG	1.47(0.98–2.20)	0.034		1.49(0.97–2.23)	0.047	
Gender							
Male	TT GT/GG	1		0.053	1		0.068
		0.90(0.61–1.23)	0.681		0.97(0.73–1.34)	0.924	
Female	TT	1			1		
	GT/GG	4.20(2.56–4.69)	0.072		3.22(2.01–5.22)	0.064	
Smoking							
Nonsmoker	TT	1		0.350	1		0.160
	GT/GG	1.38(0.98–1.94)	0.068		1.13(0.80–1.57)	0.492	
Smoker	TT	1			1		
	GT/GG	1.48(1.03–2.14)	0.034		1.90(1.29–2.80)	0.001	
Drinking							
Nondrinker	TT	1		0.520	1		0.093
	GT/GG	0.73(0.64–1.12)	0.335		0.87(0.68–3.73)	0.565	
Drinker	TT	1			1		
	GT/GG	0.81(0.66–1.28)	0.223		0.94(0.71–3.92)	0.324	

*CAD*, coronary artery disease; *IS*, ischemic stroke

### Related risk factors for CAD and IS

Multivariate logistic analysis showed that the incidence of CAD and IS positively correlated with smoking, BMI and rs9534275 GT/GG genotypes and negatively correlated with the alcohol consumption. In the meantime, the hyperlipidemia was positively correlated with CAD and hypertension was also positively correlated with IS (Table 4).

**Table 4:** The relative risk factors for CAD and IS

Relatives factors	CAD		IS	
	OR (95%CI)	*P*	OR (95% CI)	*P*
Nonsmoking	1		1	
Smoking	1.92(1.45–2.51)	0.013	1.80(1.36–2.39)	0.022
Nondrinking	1		1	
Drinking	0.29(0.22–0.38)	0.006	0.34(0.26–0.41)	0.010
BMI < 24Kg/m^2^	1		1	
BMI ≥ 24Kg/m^2^	2.07(1.62–2.65)	0.018	1.47(1.14–1.90)	0.031
Rs9534275 TT	1		1	
Rs9534275 GT/GG	1.47(1.13–1.92)	0.004	1.46(1.12–1.90)	0.005
Non-diabetes	1		1	
Diabetes	1.08(0.81–1.45)	0.466	1.28(0.96–1.70)	0.092
Normotensive	1		1	
Hypertension	1.11(0.84–1.45)	0.608	1.55(1.19–2.02)	0.010
Normal blood lipids	1		1	
Hyperlipidemia	2.48(1.87–3.29)	0.004	2.18(1.64–2.90)	0.008

*CAD*, coronary artery disease; *IS*, ischemic stroke

### Genotypes and serum lipid levels

As shown in Table 5, the TC and ApoB levels in the CAD patients were different among the TT, GT and GG genotypes (*P* = 0.023 and *P* = 0.043; respectively), the G allele carriers had higher TC and ApoB levels than the G allele non-carriers (*P* = 0.018 and *P* = 0.031; respectively).

**Table 5:** Association of the genotypes and serum lipid levels in controls and CAD and IS patients

Genotype	n	TC (mmol/L)	TG (mmol/L)	HDL-C (mmol/L)	LDL-C (mmol/L)	ApoA1 (g/L)	ApoB (g/L)	ApoA1/ApoB
Control								
TT	203	4.58 ± 0.71	1.53 ± 2.38	1.90 ± 0.52	2.80 ± 0.75	1.43 ± 0.31	0.91 ± 0.22	1.65 ± 0.54
GT	310	4.46 ± 0.50	1.37 ± 1.18	1.85 ± 0.42	2.74 ± 0.70	1.40 ± 0.23	0.90 ± 0.19	1.64 ± 0.51
GG	134	4.48 ± 0.49	1.38 ± 1.70	1.98 ± 0.54	2.72 ± 0.62	1.42 ± 0.27	0.91 ± 0.26	1.64 ± 0.46
*P*		0.070	0.580	0.058	0.530	0.444	0.629	0.941
TT	203	4.58 ± 0.71	1.53 ± 2.38	1.90 ± 0.52	2.80 ± 0.75	1.43 ± 0.31	0.91 ± 0.22	1.65 ± 0.54
GT + GG	444	4.62 ± 0.49	1.47 ± 1.36	1.89 ± 0.46	2.75 ± 0.99	1.40 ± 0.25	0.92 ± 0.26	1.63 ± 0.49
*P*		0.072	0.297	0.786	0.278	0.283	0.374	0.729
CAD								
TT	150	4.55 ± 1.41	1.66 ± 1.09	1.13 ± 0.30	2.88 ± 0.75	1.07 ± 0.86	0.90 ± 0.27	1.23 ± 0.48
GT	316	4.58 ± 2.78	1.70 ± 1.11	1.16 ± 0.34	2.92 ± 1.02	1.04 ± 0.38	1.63 ± 9.16	1.21 ± 0.52
GG	140	4.61 ± 3.61	1.64 ± 1.03	1.16 ± 0.36	3.01 ± 1.33	1.03 ± 0.34	1.54 ± 4.43	1.17 ± 0.50
*P*		0.023	0.834	0.652	0.710	0.738	0.043	0.565
TT	150	4.55 ± 1.41	1.66 ± 1.09	1.13 ± 0.30	2.88 ± 0.75	1.07 ± 0.86	0.90 ± 0.27	1.23 ± 0.48
GT + GG	456	4.72 ± 6.38	1.78 ± 1.39	1.15 ± 0.34	2.95 ± 0.99	1.04 ± 0.37	1.62 ± 5.52	1.20 ± 0.51
*P*		0.018	0.810	0.356	0.887	0.443	0.031	0.548
IS								
TT	140	4.60 ± 1.46	1.90 ± 2.23	1.26 ± 0.54	2.90 ± 0.87	1.02 ± 0.24	0.99 ± 0.22	1.12 ± 0.56
GT	291	4.52 ± 1.02	1.66 ± 1.22	1.21 ± 0.35	2.94 ± 0.83	1.03 ± 0.33	1.06 ± 0.19	1.09 ± 0.51
GG	138	4.52 ± 1.07	1.62 ± 1.21	1.24 ± 0.33	2.93 ± 0.84	1.05 ± 0.22	1.03 ± 0.26	1.09 ± 0.46
*P*		0.777	0.232	0.456	0.930	0.896	0.789	0.713
TT	140	4.60 ± 1.46	1.90 ± 2.23	1.26 ± 0.54	2.90 ± 0.87	1.02 ± 0.24	0.99 ± 0.22	1.12 ± 0.56
GT + GG	337	4.58 ± 1.38	1.65 ± 1.39	1.22 ± 0.48	2.94 ± 0.99	1.06 ± 0.62	1.04 ± 0.26	1.09 ± 0.51
*P*		0.478	0.090	0.278	0.705	0.971	0.702	0.412

Adjusted for sex, age, smoking, drinking, BMI, diabetes, hypertension, hyperlipidemia. *TC*, total cholesterol; *TG*, triglyceride; *HDL-*C, high-density lipoprotein cholesterol; *LDL-C*, low-density lipoprotein cholesterol; *ApoA1*, apolipoprotein A1; *ApoB*, apolipoprotein B

## Discussion

With the remarkable improvement of social living standard, the development of CAD was influenced by both genetic and environmental factors, as evident by its high heritability (40–50%), shown in twin and family studies [24]. Hypertension [25], obesity [26], abdominal fat [27], diabetes [28], dyslipidemia [29–31], inflammation as reflected by high levels of C-reactive protein (CRP) [32], are associated with CAD. The present study shows that the genotypic and allelic frequencies of the rs9534275 SNP were significantly different between the patients with CAD or IS and controls, and the GG, GT/GG genotypes and G allele were associated with an increased risk of CAD and IS. That was to say, the rs9534275 SNP would be a genetic factor contribute to CAD and IS.

In a previous association study, the *BRCA* mutations were found in about 20% of all hereditary breast cancers and women carrying *BRCA1* and *BRCA2* mutation were easily caught up with breast cancer [33]. Hsu et al. had found that lipids are recognized to play a crucial role in tumor development and progression, especially in breast cancer [34]. It has been showed that hypercholesterolemia increased the enzymatic formation of the oxysterol 27-OHC and accelerated tumor formation and progression in murine breast cancer models [35]. Hypercholesterolemia, not only could it cause cancer, but also it can lead to atherosclerosis which the common pathophysiologic mechanisms of CAD and IS [36]. In the current study, we found that the G allele carriers had higher TC and ApoB levels in CAD, that would be another promising aspect to increase the risk of CAD for *BRCA2* rs9534275 SNP.

As taken several environment exposures into consideration, an increased risk of CAD and IS in subjects with a GT/GG genotype was mainly noted in those with high BMI or smokers. Bangalore et al. found that fluctuation in body weight was associated with higher mortality and a higher rate of cardiovascular events independent of traditional cardiovascular risk factors [37]. Several studies have demonstrated that obesity is a common risk factor for several subtypes of cardiovascular disease, including CAD, stroke, and heart failure [38–41]. Besides, some previous researches found that smoking altered serum lipid profiles, as characterized by increased TC, TG, LDL-C levels and the ApoB/ApoA1 ratio, along with decreased HDL-C levels. These changes would regulate the risk of CAD and IS [42–44]. In our present study, the interaction between *BRCA2* rs9534275 SNP and high BMI or smoking, and an increased risk of CAD and IS were also discovered.

Nowadays, the clinical beneficial effects of therapy in reducing the risk of coronary events and mortality in patients with CAD or IS are believed to be the result of its cholesterol-lowering actions [45], the quest for pharmacologic agents that target in treating atherogenesis has intensified in recent years. In the present study, we showed that the *BRCA2* rs9534275 SNP not only modified serum lipid levels and the risk of CAD and IS, but also interacted with environment exposures. Thus, the *BRCA2* rs9534275 SNP may be a promising drug target for therapeutic intervention against hyperlipidemia and atherosclerosis.

There are several potential limitations in the present study. Firstly, many patients were taking medications such as lipid-lowering drugs (statins or fibrates), ACE inhibitors, beta blockers, diuretics, aspirin, and anti-atherosclerotic drugs. All of these drugs have different effects on serum lipid levels. Secondly, the mean values of height, weight, BMI and blood pressure were higher and the percentage of subjects who consumed alcohol was lower in CAD/IS patients than in controls. Although some factors such as sex, age, BMI, blood pressure, alcohol consumption, and cigarette smoking have been adjusted for the statistical analysis, the influence of these factors on serum lipid levels was not excluded completely. Finally although we found that the rs9534275 G allele was associated with an increased concentration of serum TC and ApoB, and also the risk of CAD and IS, we did not clarify the mechanism so that more experiments should be carried out.

## Conclusions

The present study shows that the genotypic and allelic frequencies of the rs9534275 SNP were significantly different between the patients with CAD or IS and controls. Subjects with GG genotype or G allele were associated with an increased risk of CAD and IS in smokers and subjects with a BMI ≥ 24 kg/m^2^. The GT/GG genotypes were also associated with increased serum TC and ApoB in CAD. These results suggest that the rs9534275 G allele was associated with increased serum TC and ApoB in CAD and with an increased risk of CAD and IS.

## Abbreviations

ANCOVA: Analysis of covariance
Apo: Apolipoprotein
BMI: Body mass index
GWAS: Genome-wide association study
HDL-C: High-density lipoprotein cholesterol
HWE: Hardy-Weinberg equilibrium
LDL-C: Low-density lipoprotein cholesterol
PCR: Polymerase chain reaction
RFLP: Restriction fragment length polymorphism
SNP: Single nucleotide polymorphism
TC: Total cholesterol
TG: Triglyceride
